# Elimination of *Calm1* long 3′-UTR mRNA isoform by CRISPR–Cas9 gene editing impairs dorsal root ganglion development and hippocampal neuron activation in mice

**DOI:** 10.1261/rna.076430.120

**Published:** 2020-10

**Authors:** Bongmin Bae, Hannah N. Gruner, Maebh Lynch, Ting Feng, Kevin So, Daniel Oliver, Grant S. Mastick, Wei Yan, Simon Pieraut, Pedro Miura

**Affiliations:** 1 Department of Biology, University of Nevada, Reno, Nevada 89557, USA; 2 Department of Physiology and Cell Biology, University of Nevada, Reno School of Medicine, Reno, Nevada 89557, USA

**Keywords:** 3′-UTR, calmodulin, alternative polyadenylation, mRNA localization, axon, dorsal root ganglion, hippocampus

## Abstract

The majority of mouse and human genes are subject to alternative cleavage and polyadenylation (APA), which most often leads to the expression of two or more alternative length 3′ untranslated region (3′-UTR) mRNA isoforms. In neural tissues, there is enhanced expression of APA isoforms with longer 3′-UTRs on a global scale, but the physiological relevance of these alternative 3′-UTR isoforms is poorly understood. *Calmodulin 1* (*Calm1)* is a key integrator of calcium signaling that generates short (*Calm1-S*) and long (*Calm1-L*) 3′-UTR mRNA isoforms via APA. We found *Calm1-L* expression to be largely restricted to neural tissues in mice including the dorsal root ganglion (DRG) and hippocampus, whereas *Calm1-S* was more broadly expressed. smFISH revealed that both *Calm1-S* and *Calm1-L* were subcellularly localized to neural processes of primary hippocampal neurons. In contrast, cultured DRG showed restriction of *Calm1-L* to soma. To investigate the in vivo functions of *Calm1-L*, we implemented a CRISPR–Cas9 gene editing strategy to delete a small region encompassing the *Calm1* distal poly(A) site. This eliminated *Calm1-L* expression while maintaining expression of *Calm1-S*. Mice lacking *Calm1-L* (*Calm1^ΔL/ΔL^*) exhibited disorganized DRG migration in embryos, and reduced experience-induced neuronal activation in the adult hippocampus. These data indicate that *Calm1-L* plays functional roles in the central and peripheral nervous systems.

## INTRODUCTION

Alternative cleavage and polyadenylation (APA) is the process by which a pre-mRNA is cleaved and polyadenylated at two or more polyadenylation [poly(A)] sites. This commonly results in two (or more) mRNAs with the same protein coding sequence but different length 3′-UTR. APA is pervasive, occurring in ∼51%–79% of mammalian genes (Hoque et al. 2013; Lianoglou et al. 2013). The 3′-UTR is a major target for posttranscriptional regulation via microRNAs (miRNAs) and RNA binding proteins (RBPs); thus, harboring a longer 3′-UTR can theoretically confer additional regulatory opportunities for transcripts (Sandberg et al. 2008; Mayr and Bartel 2009). Long 3′-UTRs impact translation in a cell context-specific manner (Floor and Doudna 2016; Blair et al. 2017), and elements located in alternative 3′-UTRs can influence mRNA localization in neurons (Tushev et al. 2018). Thousands of genes in mouse and human express long 3′-UTR mRNA isoforms in brain tissues (Miura et al. 2013). For many genes, short 3′-UTR isoforms were found to be expressed across various tissues, whereas the alternative long 3′-UTR isoforms were abundant only in brain. Our understanding of the in vivo functions of neural-enriched long 3′-UTR isoforms is limited despite the large number of genes shown to be affected in *Drosophila*, Zebrafish, mice, and humans (Smibert et al. 2012; Ulitsky et al. 2012; Miura et al. 2013).

Multiple studies have examined the functions of 3′-UTRs in vivo by generating mice that either genetically delete parts of 3′-UTRs or introduce foreign poly(A) sites to cause production of mRNAs with truncated 3′-UTRs (Miller et al. 2002; Perry et al. 2012). For example, a genetic approach was implemented in mice to abolish the BDNF long 3′-UTR isoform while maintaining expression of its short mRNA counterpart via the insertion of tandem SV40 poly(A) sites downstream from the proximal polyadenylation signal. These mice displayed synaptic defects and hyperphagic obesity, ostensibly due to compromised mRNA localization to dendrites and impaired translational control (An et al. 2008; Liao et al. 2012). The recent advent of CRISPR–Cas9 gene editing has revolutionized the speed and efficiency of generating deletion mouse strains (Wang et al. 2013). This presents an exciting new opportunity for rapidly generating 3′-UTR isoform-specific knockout mice. CRISPR–Cas9 has been implemented to successfully generate mice with a deletion that removed most of the mTOR 3′-UTR (Terenzio et al. 2018). However, successful generation of an isoform-specific, long 3′-UTR knockout mouse using CRISPR–Cas9 has not been reported to date.

Calmodulin (CaM) is the primary calcium sensor in the cell (Means and Dedman 1980; Yamniuk and Vogel 2004; Sorensen et al. 2013). CaM is expressed ubiquitously but is particularly abundant in the nervous system (Kakiuchi et al. 1982; Ikeshima et al. 1993). There are three *Calmodulin* genes in mammals—*Calm1*, *Calm2*, and *Calm3*. These share an identical amino acid coding sequence, but possess unique 5′ and 3′-UTRs (SenGupta et al. 1987; Fischer et al. 1988), suggesting differences in their regulation might be conferred at the posttranscriptional level. The existence of alternative 3′-UTR mRNA isoforms generated by APA for the *Calmodulin 1* (*Calm1*) gene have been known for several decades (SenGupta et al. 1987; Nojima 1989; Ni et al. 1992; Ikeshima et al. 1993). These include mRNAs with a short 0.9 kb 3′-UTR (*Calm1-S*) and one with a long 3.4 kb 3′-UTR (*Calm1-L*). The functional significance of these alternative 3′-UTR isoforms is entirely unexplored.

Previous work has shown that altering CaM levels disrupts neuronal development in *Drosophila* and rodents (Vanberkum and Goodman 1995; Fritz and VanBerkum 2000; Kim et al. 2001; Kobayashi et al. 2015; Wang et al. 2015). CaM plays a role in guiding axon projections to create connections with other cells (Vanberkum and Goodman 1995; Fritz and VanBerkum 2000; Kim et al. 2001; Kobayashi et al. 2015). Additionally, CaM interplays with a variety of synaptic proteins to play a fundamental role in regulating signaling pathways critical in synaptic plasticity (Xia and Storm 2005). Functions in the nervous system specifically for *Calm1* have been described. Notably, targeted knockdown of *Calm1,* but not *Calm2* or *Calm3,* was found to cause major migration defects in developing hindbrain neurons (Kobayashi et al. 2015). *Calm1* mRNA has been detected in cultured rat embryonic dorsal root ganglion (DRG) axons, without distinguishing *Calm1-S* or *Calm1-L* isoforms, where it undergoes local translation to promote axon outgrowth in vitro (Wang et al. 2015). *Calm1* mRNAs, especially *Calm1-L*, were found to be expressed in dendrites of rat hippocampal neurons (Tushev et al. 2018). However, whether *Calm1-L* has a specific function in the embryonic or adult nervous system has not been addressed.

Here, we utilized CRISPR–Cas9 to generate the first mouse lines lacking expression of a long 3′-UTR mRNA isoform while maintaining normal expression of its short 3′-UTR APA counterpart. We uncovered that *Calm1-L* levels were high in the DRG and hippocampus, and its expression was largely restricted to neurons. In vivo phenotypic analysis of mutant embryos revealed disorganized DRG axon and cell body positioning, whereas adults displayed a reduction in hippocampal neuronal activation after enriched environment exposure. This novel deletion methodology has thus uncovered in vivo neuronal impairments attributed to the loss of a single long 3′-UTR mRNA isoform in mice.

## RESULTS

### *Calm1-L* expression is enriched in neurons

**FIGURE 1.**
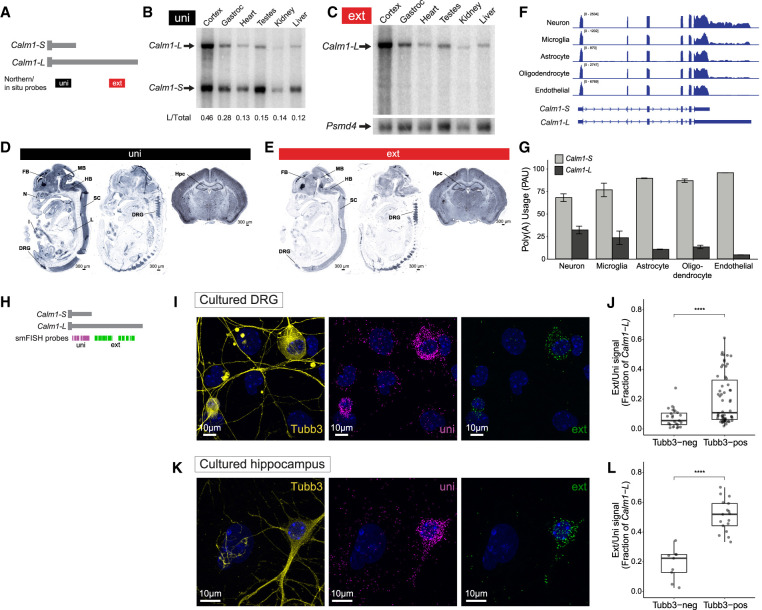
*Calm1-L* expression is enriched in neurons. (*A*) Diagram showing northern blot and DIG in situ probes to detect either both isoforms (uni) or specifically the long 3′-UTR isoform (ext). (*B*) Northern blot of an array of tissues collected from adult mice probed with uni probes showing *Calm1-S* (*bottom* arrow) and *Calm1-L* (*top* arrow). The ratio of *Calm1-L* normalized to total *Calm1* (sum of *Calm1-S* band intensity and *Calm1-L*) is shown. (*C*) Northern blot performed with ext probe. The same blot was stripped and reprobed for the housekeeping gene *Psmd4* as a loading control. (*D*) DIG in situ hybridization of E13.5 embryo and 8w brain using the uni probe showed universal expression of *Calm1* showing strong signal in the nervous system including forebrain (FB), midbrain (MB), hindbrain (HB), spinal cord (SC), dorsal root ganglion (DRG), cortex, and hippocampus (Hpc). (*E*) DIG in situ performed with the ext probe showed neural tissue-specific expression pattern of *Calm1-L* with particularly strong signals in the DRG and Hpc. (*F*) RNA-seq tracks from purified neurons, microglia, astrocytes, newly formed oligodendrocytes, and endothelial cells are visualized. *Calm1-L* gene model is shown as annotated and *Calm1-S* gene model was illustrated based on the sequencing read coverages. Read coverage in the long 3′-UTR is particularly high in neurons. (*G*) QAPA was used to estimate the fraction of *Calm1-S* and *Calm1-L* found in each RNA-seq data set. The poly(A) usage indicates that *Calm1-L* is indeed enriched in neurons. Two replicates for each cell type. (*H*) Diagram showing RNAscope smFISH probe locations. (*I,J*) smFISH performed for total *Calm1* (uni) or *Calm1-L* (ext) transcripts in primary DRG culture colabeled with β3 Tubulin (Tubb3) neuronal marker. Most cells showed robust uni signals while ext signals were restricted in Tubb3-positive cells. Significance was determined using a *t*-test, (****) *P* < =0.0001. *n* = 58 Tubb3-pos cells, *n* = 23 Tubb3-neg cells. (*K*,*L*) smFISH performed in primary hippocampal culture showing the same trend. Significance was determined using a *t*-test, (****) *P* < =0.0001. *n* = 17 Tubb3-pos cells, *n* = 9 Tubb3-neg cells.

To determine the relative expression of short and long *Calm1* 3′-UTR isoforms among mouse tissues, we performed northern blot analysis—an approach uniquely suited to report on the expression of alternative 3′-UTRs (Miura et al. 2014). Northern blots performed using a universal probe (uni) that detects both short and long *Calm1* 3′-UTR isoforms revealed the presence of both *Calm1-L* and *Calm1-S* across an array of adult tissues. The cerebral cortex showed greater enrichment of *Calm1-L* versus *Calm1-S* compared to non-brain tissues (Fig. 1A,B), as had been previously observed (Ikeshima et al. 1993; Miura et al. 2013). The ratio of long isoform to the sum of long and short isoforms (total *Calm1*) was found to be 0.46 in cortex, whereas in the rest of the tissues examined (gastrocnemius skeletal muscle, heart, testes, kidney, and liver) this value ranged between 0.12–0.28. Recent studies have shown that some 3′-UTRs can be cleaved to generate stable 3′-UTR transcripts that are separated from the protein coding portion of the mRNA (Kocabas et al. 2015; Malka et al. 2017; Sudmant et al. 2018). To test whether this might be the case for *Calm1*, and to confirm the connectivity of the long 3′-UTR region to the rest of the *Calm1* mRNA, the blot was stripped and reprobed with a probe specific for *Calm1-L* (ext). One band consistent with the size of *Calm1-L* was observed (Fig. 1C). Thus, the long 3′-UTR region is connected to the full-length mRNA, with no evidence of a cleaved 3′-UTR observed in the tissues examined.

We next determined the spatial expression pattern of *Calm1* 3′-UTR isoforms in mouse tissues. In situ hybridization (ISH) using Digoxigenin (DIG)-labeled probes was performed in embryonic day 13.5 (E13.5) mice and 8-wk-old brains. Using a *Calm1* universal probe (uni; Fig. 1A), strong signal was observed in the brain (including forebrain [FB], midbrain [MB], and hindbrain [HB]), spinal cord (SC), and dorsal root ganglion (DRG) in parasagittal sections of embryos. Uni signal was also strong in whole coronal sections of the adult brain including amygdala, striatum, hypothalamus, cortex, and hippocampus (Hpc) (Fig. 1D). Expression was also observed in the nasal epithelium (N), lung (L), and intestinal epithelium (I). In contrast, ISH performed with probe against the long 3′-UTR (ext; Fig. 1A) showed staining largely restricted to the brain, spinal cord, and DRG in parasagittal sections of the embryo and stronger signal in the adult Hpc compared to other brain regions (Fig. 1E). These experiments demonstrate that *Calm1-L* is enriched in neural tissues.

The global bias for enhanced expression of longer 3′-UTR isoforms in the nervous system has generally been attributed to expression in neurons (Guvenek and Tian 2018). To test if this is the case for *Calm1*, we analyzed the relative expression of the *Calm1* 3′-UTR isoforms in different brain cell types from a previously published RNA-seq data set (Zhang et al. 2014). We performed reanalysis of these data using QAPA, which allows for an estimation of relative poly(A) site usage (PAU) from RNA-seq reads (Ha et al. 2018). We analyzed the usage of *Calm1-S* and *Calm1-L* among purified neurons, microglia, astrocytes, newly formed oligodendrocytes, and endothelial cells. The coverage tracks of these data showed the highest enrichment of reads pertaining to the long 3′-UTR in neurons compared to the other cell types (Fig. 1F). Quantification using QAPA revealed neurons had the greatest usage of *Calm1-L* (32%) (Fig. 1G).

To determine the cellular expression of *Calm1* 3′-UTR isoforms in our tissues of interest, we performed branched-oligo based single molecule Fluorescence In Situ Hybridization (RNAscope smFISH) on primary cultures from DRG and hippocampus (Fig. 1H–L). Costaining with anti-β3Tubulin (Tubb3) was used to distinguish neurons from other cell types. In cultured DRG, most cells showed robust uni signal, but ext signal was only prominent in Tubb3-positive cells (Fig. 1I). Counting of uni and ext puncta indicated that *Calm1-L* is mostly enriched in Tubb3-positive neurons in DRG (Fig. 1J). Analysis of primary hippocampal cultures similarly revealed that the *Calm1* ext signal was significantly enriched in Tubb3-positive neurons (Fig. 1K,L). Together with the RNA-seq analysis of isolated brain cells, these data show that *Calm1-L* expression is enriched in neurons.

### Subcellular localization of *Calm1-L*

**FIGURE 2.**
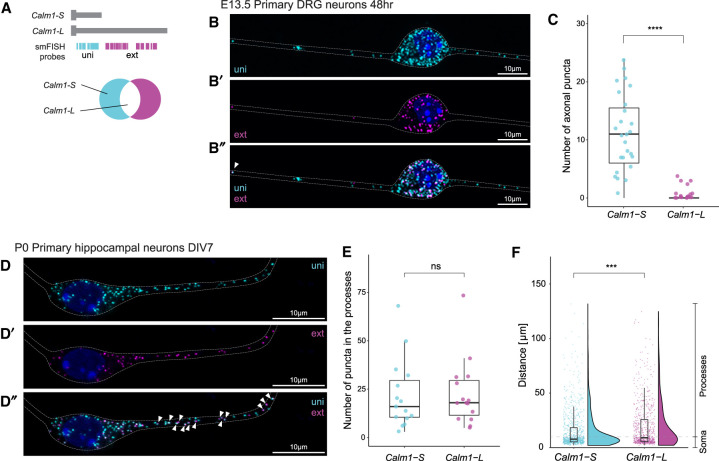
Subcellular localization of *Calm1-L*. (*A*) Diagram showing RNAscope smFISH probe locations. When uni and ext images are merged, *Calm1-L* isoforms are shown as colocalized (white) puncta. (*B*) smFISH showed robust axonal localization of *Calm1-S* (uni), but *Calm1-L* (ext/magenta in *B*′ or white punta in *B*″) was observed in DRG axons. (*C*) When the number of axonal uni or ext puncta was counted, uni signals were robustly found in the axons of DRG neurons but little to no ext signals were found in the same regions. Significance was determined using a *t*-test, (****) *P* < =0.0001. *n* = 30 neurons. (*D*) In primary hippocampal neurons, both *Calm1-S* and *Calm1-L* were observed in soma and neuronal processes. (*E*) The number of puncta corresponding to *Calm1-S* and *Calm1-L* transcript in the hippocampal processes was not found to be significantly different. Significance was determined using a *t*-test, ns: *P* > 0.05. *n* = 15 neurons. (*F*) Analysis of the distance of travel for all the *Calm1-S* and *Calm1-L* signals showed the overall distribution is different between two mRNA isoforms. Analysis done in *n* = 15 neurons (*Calm1-L* 684 puncta, *Calm1-S* 907 puncta). Significance was determined using a Wilcoxon test, (***) *P* < =0.001.

*Calm1* mRNA, without distinguishing between *Calm1-S* or -*L*, has been shown to be localized to axons and dendrites in multiple studies (Zivraj et al. 2010; Gumy et al. 2011; Kobayashi et al. 2015; Wang et al. 2015; Preitner et al. 2016; Zappulo et al. 2017; Tushev et al. 2018). Given earlier work showing that elements present in long 3′-UTRs of importin β1 and *Impa1* are involved in mRNA localization to axons (Andreassi et al. 2010; Perry et al. 2012), we hypothesized that *Calm1-L* might be specifically localized to axons. To test this hypothesis, we cultured dissociated embryonic DRG neurons in compartmentalized chambers or on glass coverslips and performed smFISH. DRG neurons are unipolar cells with a single axon stem bifurcating into a peripheral and a central branch, and use of embryonic DRG allows a high efficiency of isolating neurons (Melli and Höke 2009). Previous studies have shown total *Calm1* expression in sensory neuron axons by multiple approaches, including FISH, microarray, and RNA-seq analysis of axonal transcriptomes (Zivraj et al. 2010; Gumy et al. 2011; Wang et al. 2015; Preitner et al. 2016). In agreement with these studies, we found that the probes targeting both *Calm1* isoforms (uni) revealed signal in soma and axons (Fig. 2A,B). *Calm1-L* expression is indicated by white puncta in these experiments due to colocalization of uni (cyan) and ext (magenta) signals (Fig. 2A). We found that *Calm1-L* showed little to no signal in DRG axons (Fig. 2B′,B″). To confirm this expression pattern we reanalyzed public RNA-seq data obtained from isolated axons of DRG/spinal cord (Minis et al. 2014). QAPA analysis revealed the relative expression of the long 3′-UTR to be 23% in whole DRG. In isolated axons, there was less relative expression of the long 3′-UTR (11%) (Supplemental Fig. 1a). This result is consistent with our FISH data showing less enrichment of *Calm1-L* in axons (Fig. 2C).

A recent study that used global analysis of 3′ end sequencing found *Calm1-L* to be enriched over *Calm1-S* expression in the rat hippocampal neuropil (Tushev et al. 2018), which is a region that comprises axons, dendrites, synapses, interneurons, and glia (Cajigas et al. 2012). In order to assess subcellular localization patterns of *Calm1* 3′-UTR isoforms in mouse hippocampal neurons, we cultured postnatal day 0 (P0) mouse hippocampal neurons and performed smFISH. β3Tubulin marker was colabeled to identify neurons. We could not simultaneously monitor short and long isoforms along with multiple cellular markers (for example, both β3Tubulin and Tau to distinguish axons and dendrites) by fluorescence microscopy, thus we refer to all neuronal extensions as “processes” for this analysis. The short 3′-UTR isoform was found in both soma and processes (cyan signals in Fig. 2D″). In contrast to the results observed in DRG neurons, *Calm1-L* was found to be localized in hippocampal neuron processes (Fig. 2D′,D′′ white signals depicted with arrowheads). Quantification of the number of localized puncta per neuron and maximum distance of travel for each isoform revealed no significant difference between the two *Calm1* isoforms (Fig. 2E; Supplemental Fig. 1b). However, the distance of travel from the center of the soma out to processes was found to be significantly different between *Calm1-S* and *Calm1-L*, largely due to enriched *Calm1-S* distribution in the soma (Fig. 2F). Collectively, these results show that *Calm1-L* is not expressed outside of the soma in DRG, but is expressed in both soma and processes in hippocampal neurons.

### Generation of *Calm1* long 3′-UTR deletion mice using CRISPR–Cas9

**FIGURE 3.**
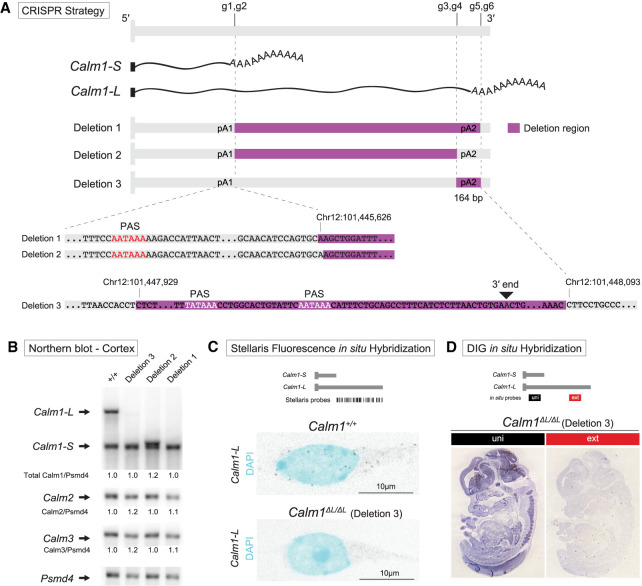
Generation of *Calm1* long 3′-UTR deletion mice using CRISPR–Cas9. (*A*) Diagram of the strategies used to eliminate the production of mature long *Calm1* 3′-UTR transcripts. Six gRNAs (g1–g6) were injected simultaneously to generate a variety of deletions (Deletion 1–3). (*B*) *Calm1* northern blot of adult cortex from three deletion strains and control littermate demonstrating successful deletion of the long *Calm1* 3′-UTR isoform. Note the Deletion 2 line generates a new isoform with a truncated long 3′-UTR due to the preservation of the distal PAS. Transcripts from the *Calm2* and *Calm3* genes were found to be unaltered. *Psmd4* used as a loading control. (*C*) Complete loss of *Calm1-L* in the *Calm1^ΔL/ΔL^* (Deletion 3) hippocampal neurons has been confirmed by lacked smFISH signals representing *Calm1-L*. (*D*) DIG in situ performed in the *Calm1^ΔL/ΔL^* (Deletion 3) embryo also revealed no expression of *Calm1-L*.

To investigate the functional role of neuron-enriched *Calm1-L* in vivo, we used CRISPR–Cas9 gene editing to generate a series of mouse lines that lacked the expression of *Calm1-L*. We aimed to generate a mutant line which did not harbor any foreign DNA sequences to promote usage of the proximal poly(A) site over the distal poly(A) site. We designed six single guide RNAs (gRNAs) targeting the *Calm1* locus that we anticipated would result in three different deletions (Fig. 3A).

Deletion mice strains were generated by injection of all six gRNAs along with mRNA encoding Cas9 endonuclease. From 11 founder mice we isolated three lines each harboring different deletions that we confirmed using Sanger sequencing. Deletion 1 removed sequence encompassing the long 3′-UTR, including the distal poly(A) site. Deletion 2 removed the majority of the sequence comprising the long 3′-UTR, save for the distal poly(A) site. This strategy brought the proximal and distal poly(A) sites adjacent to one another, which we surmised might ensure cleavage at this region and prevent selection of cryptic poly(A) sites further downstream. Deletion 3 was designed to remove the distal poly(A) site, in anticipation that although the long 3′-UTR would be transcribed, it would not be cleaved and polyadenylated, thus preventing biogenesis of mature *Calm1-L* mRNA.

The effectiveness in preventing *Calm1-L* biogenesis in these three lines was determined by northern blot analysis using the uni probe (Fig. 1A). We chose to analyze cortex samples instead of smaller tissues because a great deal of starting RNA is required to perform these mRNA northern blots. Remarkably, each deletion strategy had the desired outcome. We found that Deletions 1 and 3 exhibited complete loss of *Calm-L* transcripts (Fig. 3B). As anticipated, northern analysis of deletion 2 line showed a band migrating slightly higher than the *Calm1-S* band, indicating the biogenesis of an ectopic transcript slightly longer than *Calm1-S* due to the second poly(A) signal (PAS) (Fig. 3B). Transcripts from the *Calm2* and *Calm3* genes were also monitored by northern blot and were found to be unaltered (Fig. 3B; for full blots, see Supplemental Fig. 2).

In deciding on which line to carry out phenotypic analysis, we surmised that deletion 2 was the most confounding because an ectopic transcript was generated. The deletion 1 and 3 strategies both had the desired effect on *Calm1-L* loss without altering total *Calm1* levels. We proceeded with phenotypic analysis on the deletion 3 allele because it had the smallest amount of genomic sequence deleted (164 bp) and thus reasoned that it was the least likely to have alterations in genomic elements such as unknown enhancer elements that could be present in the long 3′-UTR-encoding region. This mouse deletion line 3 was renamed *Calm1^ΔL/ΔL^*.

We further verified the complete loss of *Calm1-L* in the *Calm1^ΔL/ΔL^* hippocampus using Stellaris smFISH and in the DRG by ISH. Cultured hippocampal neurons from the *Calm1^ΔL/ΔL^* mice completely lacked signal representing *Calm1-L* (Fig. 3C). ISH for *Calm1-L* performed in E13.5 embryo sections also revealed no expression (Fig. 3D). We confirmed by Sanger sequencing that the *Calm1^ΔL/ΔL^* line did not harbor unintended mutations at predicted gRNA off target sites (Supplemental Fig. 3).

### *Calm1^ΔL/ΔL^* mice exhibit DRG development defects

**FIGURE 4.**
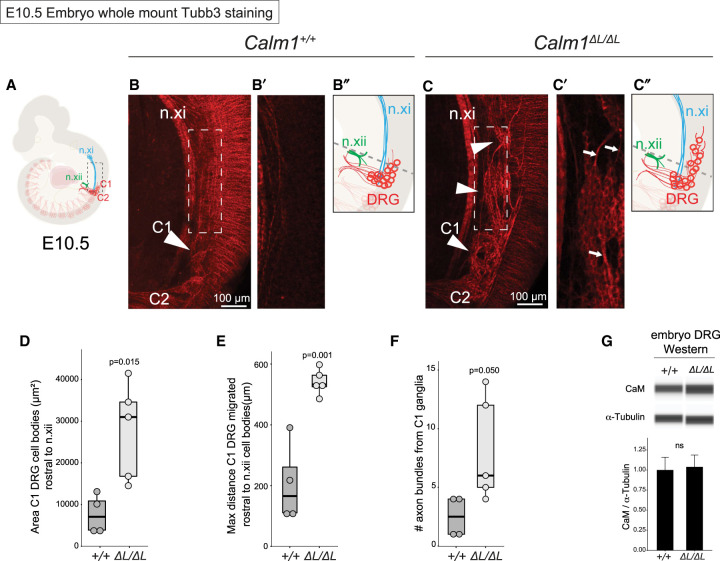
*Calm1^ΔL/ΔL^* mice exhibit DRG axon development defects. Developing C1 DRG exhibit axonal and cell body migration disorganization in *Calm1^ΔL/ΔL^* E10.5 embryos. (*A*) Schematic of *Calm1^+/+^* E10.5 embryo highlighting the morphology of the C1 and C2 DRG axons and cell bodies. (*B*,*C*) Whole mount *Calm1^+/+^* and *Calm1^ΔL/ΔL^* DRG morphology visualized by anti-Tubb3 labeling. (*B*) The cell bodies of the C1 DRG (arrowhead) in *Calm1^+/+^* embryos are bundled together to form a distinct ganglion. The neurites of the C2 DRG can be seen projecting ventrally in an organized bundle. (*C*) The C1 DRG in *Calm1^ΔL/ΔL^* animals is disorganized and consists of clusters of cell bodies (arrowheads) that extend bundles of axons (arrows). The C1 DRG cells of deletion animals migrate rostral relative to *Calm1^+/+^* and are adjacent to the n.xi tract (*B*′,*C*′). Enlarged view of single optical section of same Z-stack from *B* and *C* focusing on disorganized mutant cell body morphology of ganglion that aberrantly migrated more rostral relative to control. (*D*–*F*) In order to start measurements in the same relative location between embryos, n.xii was used as an anatomical landmark to set a beginning of measurements, indicated by the gray dashed lines in *B*″,*C*″. (*D*) Quantification of area of ectopic cell bodies observed for developing DRG. (*E*) Distance of aberrantly clustered cell bodies in *Calm1^+/+^* and *Calm1^ΔL/ΔL^*. (*F*) Quantification of the number of axon bundles projecting off C1 ganglia. Significance determined by a *t*-test; *n* = 4 C1 DRG *Calm1^+/+^*; *n* = 5 C1 DRG *Calm1^ΔL/ΔL^*. n.xi = accessory nerve, n.xii = hypoglossal nerve. (*G*) Capillary western analysis of *Calm1^+/+^* and *Calm1^ΔL/ΔL^* embryonic DRG showing no change in overall protein levels. Significance was determined using a *t*-test, *P* = 0.802; *n* = 3.

Given the pronounced expression of *Calm1-L* in DRG (Fig. 1E) we examined *Calm1^ΔL/ΔL^* embryos for DRG developmental defects. The DRG is a series of ganglia in the peripheral nervous system derived from neural crest cells, which by early embryonic stage E10.5 have already begun to form distinct ganglia adjacent to the spinal neural tube caudal to the hindbrain (Fig. 4A; Le Douarin and Smith 1988; Marmigere and Ernfors 2007). Cell bodies of the DRG send out dorsolateral axon projections concurrent with neurogenesis during this stage in development (Marmigere and Ernfors 2007). Unlike DRG that form adult structures, the first cervical (C1) DRG is a temporary embryonic population that loses its dorsolateral axonal projections and progressively undergoes programmed cell death from E10.5 to ∼E12.5 (Fanarraga et al. 1997; van den Akker et al. 1999).

The axons and cell bodies of the C1 DRG in *Calm1^ΔL/ΔL^* embryos at E10.5 were found to be severely disorganized relative to *Calm1^+/+^* embryos (Fig. 4B,C). Large groups of cell bodies of the C1 DRG in mutant embryos translocated rostral into the hindbrain adjacent to the accessory nerve (n.xi) tracks (Fig. 4C–C″). At the same location in *Calm1^+/+^* embryos, there were fewer translocating C1 DRG bodies as rostral and disorganized (Fig. 4B–B″). The C1 DRG cell bodies in *Calm1^ΔL/ΔL^* grouped together in smaller ganglia (Fig. 4C–C″). Axons branching off these cell bodies projected aberrantly and were tightly fasciculated (Fig. 4C′, see arrows). Some *Calm1^ΔL/ΔL^* axons projected longitudinally, which was in contrast to the dorsolateral projections seen in non-C1 DRG (see Materials and Methods). Significantly more C1 DRG cell bodies rostrally migrated in the mutants compared to controls (Fig. 4D,E). Lastly, we quantified the number of fascicles projecting off the C1 ganglia and found there were significantly higher numbers emanating from *Calm1^ΔL/ΔL^* DRG compared to *Calm1^+/+^* (Fig. 4F). Together these data show that loss of *Calm1-L* impairs C1 DRG axon development and restricts rostral cell migration.

We reasoned that the impairment of DRG development in *Calm1^ΔL/ΔL^* was a result of altered translation of *Calm1* resulting from loss of the long 3′-UTR isoform. We thus performed Western analysis for CaM in wild type and mutant dissected E13.5 DRG. Note that three genes encode identical CaM protein, thus this analysis cannot uniquely report on CaM generated from *Calm1*. Western analysis was performed using capillary immunoassay due to the low amounts of DRG material recovered from dissection. Western analysis failed to identify a significant change in CaM levels between *Calm1^+/+^* and *Calm1^ΔL/ΔL^* embryonic DRGs (Fig. 4G).

### Subcellular localization of *Calm1* and CaM levels are unaltered in *Calm1^ΔL/ΔL^* hippocampus

**FIGURE 5.**
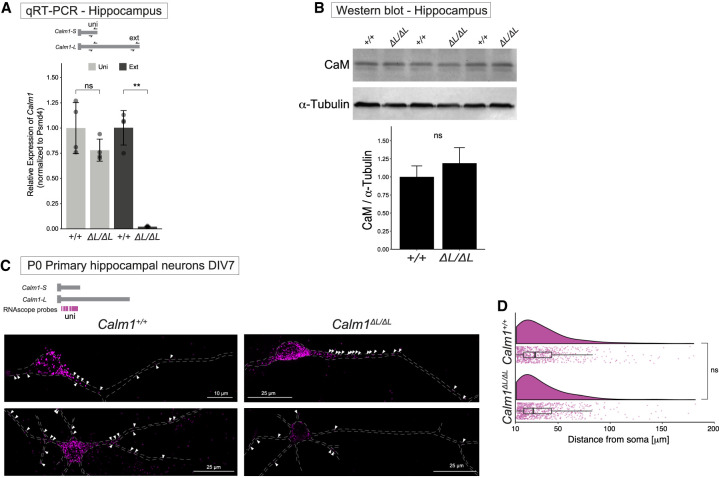
Characterization of *Calm1* isoforms in the hippocampus. (*A*) qRT-PCR analysis revealed no changes in total *Calm1* (uni) RNA levels between *Calm1^+/+^* and *Calm1^ΔL/ΔL^*, while the *Calm1-L* expression is abolished in *Calm1^ΔL/ΔL^* hippocampus. Significance was determined using a *t*-test, ns: *P* > 0.05, (**) *P* < =0.01, *n* = 4 mice. (*B*) Western blot analysis of *Calm1^+/+^* and *Calm1^ΔL/ΔL^* adult hippocampus showing no change in overall protein levels. Significance was determined using a *t*-test, *P* = 0.287; *n* = 3 mice. (*C*,*D*) smFISH analysis in both *Calm1^+/+^* and *Calm1^ΔL/ΔL^* hippocampal neurons showed that the deletion of *Calm1-L* did not significantly impair localizing potential of *Calm1* mRNAs in hippocampal neurons. Significance was determined using a Wilcoxon test, ns: *P* > 0.05; *n* = 19 *Calm1^+/+^* neurons (739 puncta), *n* = 26 *Calm1^ΔL/ΔL^* neurons (802 puncta).

Next, we examined whether *Calm1^ΔL/ΔL^* mutants experience neurological defects in the central nervous system. We directed our attention to the hippocampus given the high expression of *Calm1-L* in this tissue (Fig. 1E). First, we characterized whether the loss of *Calm1-L* transcripts altered the total amount of total *Calm1* transcripts or CaM protein in the hippocampus. qRT-PCR and western blot analysis revealed no changes in total *Calm1* RNA or CaM protein levels between *Calm1^+/+^* and *Calm1^ΔL/ΔL^* (Fig. 5A,B). Additionally, we assessed changes in *Calm1* mRNA localization into the processes of hippocampal neurons. smFISH analysis in both *Calm1^+/+^* and *Calm1^ΔL/ΔL^* hippocampal neurons showed that the deletion of *Calm1-L* did not significantly impair localization of *Calm1* mRNAs in hippocampal neurons (Fig. 5C,D). Thus, our results suggested that the overall *Calm1* mRNA levels, subcellular localization of *Calm1* mRNAs, or CaM protein levels are not significantly altered in *Calm1^ΔL/ΔL^* hippocampus compared to *Calm1^+/+^*.

### *Calm1^ΔL/ΔL^* mice exhibit reduced hippocampal IEG expression in response to enriched environment exposure

CaM is an integral part of CaMK, Calcineurin, and MAPK signaling pathways which are involved in synaptic transmission and plasticity (Xia and Storm 2005; Pang et al. 2010; Hagenston and Bading 2011; Sethna et al. 2016). Ca^2+^ signaling mediates synaptic plasticity via posttranslational alterations and initiating signaling cascades resulting in de novo transcription (Greer and Greenberg 2008; Hagenston and Bading 2011). With this in mind, we hypothesized the *Calm1-L* might impact CaM-dependent synaptic transmission and/or plasticity. As a proof of principle, we used an enriched environment (EE) paradigm to induce experience-driven expression of immediate early genes (IEGs) in *Calm1^ΔL/ΔL^* hippocampi and compared it to *Calm1^+/+^*. IEG expression, especially the canonical example of cFos induction, is a marker of neuronal activation and is considered to be required for learning and synaptic plasticity (Yap and Greenberg 2018).

**FIGURE 6.**
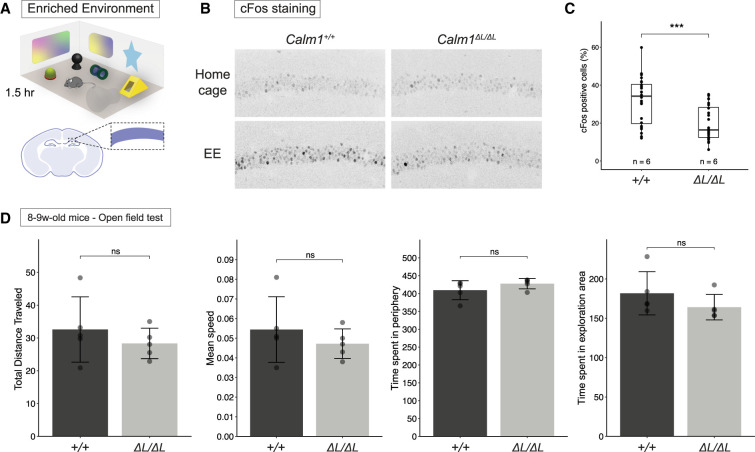
*Calm1-L* loss reduces hippocampal IEG expression in response to enriched environment exposure. (*A*) Graphic illustration of enriched environment used in this study and the CA1 region shown in *B*. (*B*) Representative images showing EE-induced expressions of cFos in *Calm1^+/+^* and *Calm1^ΔL/ΔL^* CA1. (*C*) Percentages of cFos-positive cells in whole CA1 region was quantified in FIJI/ImageJ. Significance was determined using a *t*-test, (***) *P* < =0.001. *n* = 6 mice (two hemispheres in two brain sections for each mouse). (*D*) Levels of total distance traveled, mean speed, time spent in periphery and exploration areas of *Calm1^+/+^* and *Calm1^ΔL/ΔL^* mice were compared using open field test. Significance was determined using a *t*-test in *n* = 5 mice; ns: *P* > 0.05.

Exposure to environmental novelty leads to a selective increase of cFos in the rat Cornu Ammonis 1 (CA1) region (VanElzakker et al. 2008) and novel object and place induces cFos expression in the rat CA1 (Ito and Schuman 2012). Immediately after exposing the mice to EE for 1.5 h, animals were sacrificed, and brains were collected. EE-induced expressions of cFos were compared in *Calm1^+/+^* and *Calm1^ΔL/ΔL^* CA1 (Fig. 6A–C). In home-cage control groups, <2% of cells in CA1 were cFos-positive. This is consistent with previous reports that showed cFos expression in the home cage condition is extremely low, with numerous sections containing no positive neurons (Drew et al. 2011). As expected, EE induced cFos expression in CA1 to 31.9 ± 12.5% in *Calm1^+/+^* mice (Fig. 6B,C). The EE induction of cFos expression in *Calm1^ΔL/ΔL^* mice was significantly reduced compared to wild-type controls (19.8 ± 9.1%) (Fig. 6B,C; Supplemental Fig. 4a). Analysis of Npas4, another IEG that is also activated in response to neuronal activity (Sun and Lin 2016), consistently showed reduced expression in the *Calm1^ΔL/ΔL^* hippocampus (Supplemental Fig. 4b).

The EE paradigm is influenced by multiple factors that mediate neural responses including social interaction, sensory stimulation, learning, and motor activity (Alwis and Rajan 2014). In order to exclude the possibility that the impaired locomotor function of *Calm1^ΔL/ΔL^* mice might have impacted EE induced IEG expression, we performed an open field test. Both *Calm1^+/+^* and *Calm1^ΔL/ΔL^* genotypes showed similar levels of total ambulatory distance, mean speed, time spent in periphery, and time spent in exploration area (Fig. 6D). Thus, locomotor function and exploratory behavior were not altered by loss of *Calm1-L* under these conditions.

We also performed CaM western blot of hippocampal lysates from *Calm1^+/+^* and *Calm1^ΔL/ΔL^* mice exposed to the enriched environment (Supplemental Fig. 4c) and found total CaM protein levels to be unchanged. This result was similar to what was observed in Jones et al., where total CaM level in the hippocampus was unchanged in response to novel-context exposure (Jones et al. 2018). Again, our results show that total CaM level was not altered by the deletion of *Calm1-L* or by experience-dependent neuronal activation.

### *Calm1-L* is less stable than *Calm1-S*

Unable to pinpoint the exact molecular defect responsible for the phenotypes in DRG and hippocampus resulting from *Calm1-L* loss, we turned our attention to the mRNA stability of *Calm1* 3′-UTR isoforms. A commonly used technique to estimate the half-life of mRNAs is tracing transcripts by qRT-PCR during a timeframe after blocking transcription. Due to *Calm1-S* and *Calm1-L* isoforms sharing a common short 3′-UTR region, we cannot quantify *Calm1-S* exclusively using qRT-PCR approaches in wild-type tissue. To overcome this, we used *Calm1^ΔL/ΔL^* mice to measure the stability of *Calm1-S*. Extension qRT-PCR primers (ext) were used to detect *Calm1-L* from *Calm1^+/+^* samples, and universal qRT-PCR primers (uni) were used to uniquely detect *Calm1-S* from *Calm1^ΔL/ΔL^* samples (Supplemental Fig. 5). *Hprt* was quantified as a normalizing gene (La Fata et al. 2014). Primary cortical neurons were treated with actinomycin D and samples collected immediately and at 3, 6, and 8 h posttreatment. An exponential regression equation was fitted to the relative abundance of each isoform across the timepoints as relative amount = e^−kdecay*time^ and the half-life was estimated for each transcript. *Calm1-S* was found to have a longer half-life (*t*_1/2_ = 5.9 ± 1.4 h) compared to *Calm1-L* (*t*_1/2_ = 2.6 ± 0.6 h) (Supplemental Fig. 5a). When *Calm1* transcript levels were normalized to the stable *Hprt* transcripts, *Calm1-S* was consistently found to be more stable than *Calm1-L* (Supplemental Fig. 5b). To discard the possibility that *Calm1^ΔL/ΔL^* line might have overall RNA decay/stability pathways affected due to the genomic manipulation, we also measured the total *Calm1* levels using uni primers in the *Calm1^+/+^* samples. This again showed that the total *Calm1* in *Calm1^+/+^* samples has higher stability compared to the *Calm1-L* (Supplemental Fig. 5c). Thus, we conclude that *Calm1-S* isoform has a higher half-life compared to *Calm1-L* isoform.

Since enhanced RNA secondary structure affects mRNA half-life in mammalian systems (Sun et al. 2019), we examined predicted secondary structures in the long versus short 3′-UTRs (Bellaousov et al. 2013). We calculated the length-normalized minimum thermodynamic free energy (−ΔG/nt) of predicted RNA structures and estimated the structural complexity of the 3′-UTRs in *Calm1-S* and *Calm1-L* (Supplemental Fig. 6). This analysis suggested more complex RNA structures are found in the *Calm1-L* 3′-UTR (−ΔG/nt = −0.297) compared to the *Calm1-S* 3′-UTR (−ΔG/nt = −0.248). Thus, differential RNA structure could account for mRNA stability differences between *Calm1-S* and *Calm1-L*.

## DISCUSSION

Here, we successfully implemented CRISPR–Cas9 gene editing in mice to eliminate expression of a long 3′-UTR isoform while not altering the expression of its corresponding short 3′-UTR isoform. To our knowledge, this is the first successful implementation of such an approach specifically for a long 3′-UTR isoform in vivo. We found that elimination of the *Calm1* long 3′-UTR isoform impaired both development of the DRG in embryos and activation of adult hippocampal neurons, thus establishing a functional role for *Calm1-L* in the peripheral and central nervous systems. Our method for long 3′-UTR deletion using CRISPR–Cas9 adds an important new tool for the characterization of APA-generated long 3′-UTR transcript isoforms.

Despite the prevalence of alternative length 3′-UTR isoforms in metazoan genomes, few physiological roles for these transcripts using loss-of-function genetic approaches have been identified. In a previous study, an in vivo neurological function for the *Bdnf* long 3′-UTR isoform was identified by generating a transgenic mouse that had tandem SV40 poly(A) sites inserted downstream from the proximal poly(A) signal to prevent biogenesis of the long 3′-UTR (An et al. 2008). Our strategy for generating loss of long 3′-UTR isoforms is less confounding because artificial regulatory sequences are not inserted into the genome. Including these strong regulatory sequences generates chimeric short 3′-UTR transcripts, which can affect cleavage and polyadenylation dynamics in unexpected ways.

We used multiple gRNAs for CRISPR–Cas9 gene editing because it was not clear which, if any, of our deletion strategies would effectively prevent *Calm1* long 3′-UTR biogenesis. One concern was that removal of the distal poly(A) site might result in activation of downstream cryptic poly(A) sites. Remarkably, a single injection of this gRNA cocktail led to three different deletions which all prevented *Calm1-L* expression. We used mice generated by deletion strategy #3 for our analysis because this completely eliminated *Calm1-L* expression and only removed 164 bp of sequence encompassing the distal poly(A) site. However, the other two deletion strategies might be worth considering for removing long 3′-UTR isoforms for other genes. For example, one advantage of deletion #1 strategy [which eliminates most of the long 3′-UTR and distal poly(A) site] is that it leaves no possibility for generating the long 3′-UTR isoform. However, implementing CRISPR for eliminating 3′-UTRs of extreme lengths (>10 kb) is likely to be of very low efficiency. Deletion strategy #2 removes the sequence in between the proximal and distal poly(A) sites. The major confounding factor for this strategy is that an ectopic, truncated mRNA slightly longer than the short isoform is produced due to the distal poly(A) site remaining intact. Still, this approach could be useful because bringing two adjacent poly(A) sites in proximity is likely to prevent usage of downstream cryptic poly(A) sites.

Genome-wide analyses consistently have found *Calm1* to be subcellularly localized in neurons (Gumy et al. 2011; Tushev et al. 2018). In accordance with these data, our smFISH experiments show *Calm1* mRNA subcellular localization to processes in both DRG and hippocampus. A well characterized example of a gene with different subcellular localizations of 3′-UTR isoforms is *Bdnf*. *Bdnf-L* is dramatically enriched in dendrites compared to the soma restricted *Bdnf-S*, and removal of the long 3′-UTR isoform was found to prevent mRNA localization into dendrites (An et al. 2008). We had hypothesized that the long 3′-UTR of *Calm1* might serve a similar role—however, smFISH experiments revealed that both the short and long 3′-UTR isoforms were expressed in the processes of hippocampal neurons, and only the short 3′-UTR isoform was found in DRG axons (Fig. 2). Thus, *cis*-elements uniquely found in the long 3′-UTR of *Calm1* do not drive the axon/dendrite localization of *Calm1* mRNAs. Along these lines, recent genome-wide analysis of transcript isoform localization in somata versus neuropil similarly did not uncover a bias for longer 3′-UTR APA isoforms to be particularly localized to neuropil (Tushev et al. 2018).

Neural development requires execution of precise gene regulatory programs in response to extracellular cues, which results in cytoskeletal rearrangements allowing for the formation of neural circuits (Tessier-Lavigne and Goodman 1996). In recent years, gene regulation at the posttranscriptional level has been emerging as an important aspect of axon growth and guidance (Holt and Schuman 2013; Zhang et al. 2019a,b). In particular, there are many examples of local translation occurring in the developing growth cone in response to extracellular cues (Campbell and Holt 2001; Lin and Holt 2007; Zivraj et al. 2010; Shigeoka et al. 2013). Our data suggests that *Calm1-L* is important for C1 DRG cell body migration and axon extensions during development. This was evident from in vivo experiments that displayed dramatically disorganized cell bodies and abnormal axonal extensions in the C1 population of DRG in *Calm1^ΔL/ΔL^* embryos (Fig. 4). Future studies are needed to determine how *Calm1-L* is involved in DRG cell body positioning and axon outgrowth given that we did not observe a role for altered CaM protein nor altered axonal localization of *Calm1-L*.

EE-induced IEG expression was impaired in the hippocampus of our *Calm1^ΔL/ΔL^* mice. IEGs play key roles in synaptic plasticity, social and cognitive functions (Coutellier et al. 2012; Chung 2015). It has been shown that blocking CaM activity inhibits long-term potentiation (Malenka et al. 1989), supporting a critical role of CaM in synaptic plasticity. It is thus possible that loss of *Calm1-L* in *Calm1^ΔL/ΔL^* mice impairs synaptic plasticity and/or cognitive functions. Despite the impairment in IEG activation, we found that CaM levels were unchanged in the hippocampus of *Calm1^ΔL/ΔL^* mice. It is possible that *Calm1* is not regulated by activity-induced local translation. Alternatively, CaM translational changes resulting from *Calm1-L* loss might be restricted to certain subcellular regions, such as the activated synapse. Indeed, the activity-induced translation of *Calm1* has been supported by synaptoneurosome polysome fractionation experiments which showed a very small but significant enrichment of *Calm1* mRNAs in the heavy-polysome fraction after NMDAR-activation via NMDA and glutamate (Jones et al. 2018). Further investigation into the role of *Calm1-L* in activity-induced synaptic translation of *Calm1* would be enabled by generating a mouse harboring an epitope tag fused to the *Calm1* coding sequence in the *Calm1^+/+^* and *Calm1^ΔL/ΔL^* backgrounds. Combined with spatiotemporal analysis of translation using a method such as Puro-PLA (Tom Dieck et al. 2015), such a transgenic line could be used to determine how *Calm1-L* 3′-UTR loss impacts activity-induced CaM translation specifically from the *Calm1* locus in vivo.

Finally, it is worth considering that the long 3′-UTR of *Calm1-L* might not be particularly important for *cis-*regulation of *Calm1* translation. Instead of impacting translation of their host genes, some long 3′-UTR isoforms act as scaffolds for assembling proteins that later form complexes with the protein being translated (Brigidi et al. 2019; Lee and Mayr 2019). Long 3′-UTRs have noncoding functions that are completely removed from the functions of the encoded protein. For example, long 3′-UTR isoforms of *Ube3a1* function as a miRNA sponge in the synaptodendritic regions independent of its protein-coding ability, and a nontranslatable long 3′-UTR isoform of *Tp53inp2* is involved in TrkA receptor internalization in axons (Valluy et al. 2015; Crerar et al. 2019).

The mechanism of how *Calm1* long 3′-UTR isoform loss impairs neuronal development and function remains unclear. Nonetheless, our approach has uncovered neural phenotypes both in the PNS and CNS resulting from the loss of a long 3′-UTR isoform in mice. We have demonstrated an effective strategy for eliminating long 3′-UTR isoforms via CRISPR/Cas9 gene editing without affecting short 3′-UTR expression. Thousands of alternative long 3′-UTR isoforms documented in mice are of unknown physiological relevance (Miura et al. 2013). The CRISPR approach described here can be used to generate deletion strains on a gene-by-gene basis to determine which long 3′-UTR isoforms have in vivo functional relevance.

## MATERIALS AND METHODS

### Animal use and tissue collection

All mice were housed in an environmentally controlled facility under the supervision of trained laboratory support personnel. Animal protocols were approved by the University of Nevada, Reno Institutional Animal Care and Use Committee (IACUC) and in accordance with the standards of the National Institutes of Health Guide for the Care and Use of Laboratory Animals.

For embryo collection, crossed female mice were monitored daily for vaginal plugs, with positive identification being counted as E0.5 at noon that day. Pregnant mice were euthanized at noon using CO_2_ asphyxiation, and then cervical dislocation was performed. E10.5 and E13.5 embryos were extracted in PBS. Embryos were immediately used for fresh tissue collection or fixed in 4% PFA in PBS by immersion. Postnatal day 0 (P0)–P1 pups were euthanized by decapitation and tissues were collected in cold PBS or HBSS. For adult tissue RNA or protein extraction, dissected tissue was flash frozen in liquid nitrogen and stored at −80°C or immediately used. To obtain fixed tissues, 7- to 9-wk-old animals were euthanized by overdose of isoflurane inhalation followed by transcardial perfusion with abundant PBS and 4% PFA in PBS.

### RT-qPCR and northern analysis

Flash frozen tissue was pulverized using a Cellcrusher tissue pulverizer. RNA was then extracted using the RNeasy Plus Universal Mini Kit (Qiagen) or TRIzol (Qiagen) method and quantified using a NanoDrop spectrophotometer. For RT-qPCR, 1 or 2 µg of RNA was reverse transcribed using SuperScript III Reverse Transcriptase (Invitrogen) or Maxima Reverse Transcriptase (Invitrogen). The cDNA reaction was diluted fivefold in ultrapure water for use in RT-qPCR. RT-qPCR was performed using SYBR Select Master Mix for CFX (Applied Biosystems). The BioRad CFX96 real time PCR machine was used to carry out real time PCR and results were analyzed using the delta-delta CT method. For northern analysis, poly(A)^+^ RNA was extracted from total RNA using NucleoTrap mRNA kit (Machery-Nagel). Northern blot analysis was performed as previously described (Miura et al. 2013). Briefly, poly(A)^+^ RNA samples (2 µg) were denatured in glyoxal and run in BPTE gels prior to downward transfer followed by northern blotting using 32-P dCTP labeled DNA probes (sequences of primers used to generate probes are found in Supplemental Table 1). Blots were exposed overnight until desired intensity of signals was detected using Typhoon FLA7000 phosphoimager (GE).

### Digoxigenin in situ hybridization

Riboprobes were generated via in vitro transcription using DIG RNA labeling mix (Roche) for the same probe regions as in northern blot analysis. Sucrose cryoprotected E13.5 embryos or adult brains were embedded in O.C.T compound and cryosectioned at 16–18 µm. Sections were treated in antigen retrieval solution for 5 min in 95°C water bath and washed in water twice. To aid permeabilization, slides were immersed in ice-cold 20% (v/v) acetic acid for 20 sec. Upon dehydration in sequential washes in 70%–100% ethanol, slides were stored at −20°C until use. Approximately 30 ng riboprobes were used in each hybridization at 65°C overnight. Stringent washes were performed in SSC buffer (50% formamide in 2× SSC and 0.5× SSC). Anti-DIG-AP Fab antibody (Roche) incubation was performed in 2% BSA in MABT solution for 1 h at room temperature. Color development was carried out using NBT and BCIP (Roche) at room temperature until desired coloring was observed (between 16 to 20 h). Leica DM IL LED microscope or Keyence BZ-X710 all in one fluorescence microscope was used for imaging and stitched automatically.

### RNA-seq analysis for 3′-UTR alternative polyadenylation identification

RNA-seq data sets for neuron, microglia, astrocyte, oligodendrocyte, and endothelial cells were downloaded from GEO accession series GSE52564, and data sets for DRG and isolated axons from GSE51572. Data sets were aligned and processed using HISAT2, Samtools, and Bamtools. Bam or bedgraph files were loaded into IGV for track visualization. For 3′-UTRs alternative polyadenylation analysis, 3′-UTRs reference was built according to QAPA algorithm with the exception of slight modification for *Calm1* 3′-UTRs. The original QAPA reference contained 6 *Calm1* 3′-UTR isoforms, but unconfirmed shorter isoforms were omitted for accurate quantification of only *Calm1-S* and *Calm1-L* expressions. Isoform abundance was quantified using Sailfish and QAPA quant algorithms. PAU values from the output file were used for comparison of 3′-UTR expression in each cell type.

### Primary neuron culture

DRGs from E13.5 mouse embryos were dissected in Neurobasal medium. Cells were dissociated with 0.25% trypsin and triturated with a gel-loading tip. Dissociated cells were plated on PDL and laminin coated compartmentalized chamber (Xona microfluidics, XC450) or coverslips in culture medium (DMEM supplemented with 1× Glutamax, 10% FBS, 25 ng/mL NGF, and 1× pen/strep). In particular for the compartmentalized chamber, the somal compartment was coated only with Poly-d-lysine (PDL) and supplemented with medium containing 10 ng/mL NGF instead of 25 ng/mL to promote the growing of axons through the microgroove and toward the axonal compartment. The cultures were maintained until DIV2 at 37°C with 5% CO_2_.

Cortices or hippocampi from P0–P1 mouse pups were dissociated with 0.25% trypsin and plated in plating media (MEM supplemented with 0.5% w/v glucose, 0.2 mg/mL NaHCO_3_, 0.1 mg/mL transferrin, 10% FBS, 2 mM l-glutamine, and 0.025 mg/mL Insulin) onto PDL coated six-well plates. After 1 d in vitro (DIV), the media was replaced with growth media (hippocampal-MEM supplemented with 0.5% w/v glucose, 0.2 mg/mL NaHCO_3_, 0.1 mg/mL transferrin, 5% FBS, 0.5 mM l-glutamine, and 2% B-27 supplement; cortical-Neurobasal media supplemented with 2% B27 supplement and 1× GlutaMax). An amount of 4 µM AraC (Sigma-Aldrich) was added to the growth media at DIV1–2 to suppress the growth of nonneuronal cells and enrich postmitotic neurons. The cultures were maintained until DIV7 at 37°C with 5% CO_2_.

### Single molecule fluorescence in situ hybridization

Single molecule FISH (smFISH) was performed in primary neurons using Stellaris (LGC Biosearch Technologies) or RNAscope multiplex fluorescent assay according to manufacturer's instruction. Briefly, Stellaris smFISH was carried out according to manufacturer's protocol for adherent cells except that Wash buffer A was replaced with 2× SSC and 10% formamide in ultrapure water, Hybridization buffer with 2× SSC, 10% formamide, 10× dextran-sulfate, and 250 nM probe. Hybridization was carried out for ∼18–24 h in the dark at 37°C. For RNAscope smFISH, cells were pretreated with permeabilization solution and protease solution, and incubated with 1× probe solution (551281-C3 or C1 for extension probe and 556541-C2 for universal probe diluted in probe diluent) for 2 h at 40°C. Subsequently, Amp1-FL to Amp4-FL were hybridized to amplify FISH signals. To combine FISH and immunofluorescence, immunostaining procedure was performed after the Amp4-FL hybridization step. Anti-Tubb3 primary and secondary antibodies were incubated for 1 h at 37°C and RT, respectively. Samples were counterstained with DAPI and mounted in anti-fade buffer (10 mM Tris pH 8.0, 2× SSC, and 0.4% glucose in water) or ProLong diamond antifade mountant (Invitrogen). During image acquisition, control neuron slides that had been incubated in probe diluent without probes were used to set laser intensity to ensure no background signal was counted in experimental conditions. Laser intensity and detector range were kept constant among different conditions. FISH-quant (Mueller et al. 2013) was used for puncta counting of FISH signals and FIJI/ImageJ was used to measure the distance of travel of each FISH signal from the center of the nucleus.

### RNA stability assays and structure predictions

Cortical neurons were treated with 1 µg/mL Actinomycin D at DIV6. Total RNA was collected in TRIzol at 0, 3, 6, and 8 h posttreatment. An amount of 1 µg of RNA was reverse transcribed using Maxima Reverse Transcriptase (Invitrogen). RT-qPCR was performed as described above. To compare the relative stability of *Calm1-S* and *Calm1-L* isoforms, expression levels of each transcript for each time point were calculated relative to 0 h using BioRad CFX Manager software and normalized to *Hprt* (a stable transcript control). To estimate the half-life of each transcript, expression level for each time point was calculated relative to 0 h without normalizing to *Hprt*. Exponential regression equations were fitted for each degradation plot. Half-life of each transcript was calculated using Goal-seek function in Excel to find the time point where the relative amount of transcript was reduced to 50%. The half-life of each biological replicate was used to determine mean and standard deviation for each transcript.

RNA structure prediction was performed in RNAstructure Web server (Bellaousov et al. 2013) with default setting and −ΔG/nt was calculated using the energy value given from the analysis and the length of the 3′-UTR (Fischer et al. 2020).

### Western analysis

For conventional western blot analysis, total protein was extracted in RIPA buffer supplemented with protease inhibitor tablet (Pierce). Protein samples were separated in 15% discontinuous SDS-PAGE gel and transferred onto 0.2 µm PVDF membrane (Trans-Blot Turbo, BioRad). Membranes were blocked in 5% skim milk followed by overnight incubation with primary antibodies at 4°C. Anti-CaM (Abcam 45689) and anti-α-tubulin (Sigma T9026) antibodies were used at 1:500–1000 and 1:2000, respectively. HRP conjugated secondary antibody incubation was performed at room temperature for 1 h. HRP signal was detected using ProSignal Femto reagent (Prometheus) and imaged using a ChemiDoc Touch (Biorad).

For low-input western blot analysis, capillary western system (Wes, Protein simple) was used. A total of 500 ng of total protein lysate was loaded per capillary to visualize proteins of interest. Anti-CaM and anti-α-tubulin were used at 1:50 dilution. Quantification of band intensity was automatically estimated using Compass for SW software.

### CRISPR–Cas9 gene editing

The MIT CRISPR Design Tool (http://www.genome-engineering.org/crispr) was used to design gRNAs targeted to various regions of the *Calm1* long 3′-UTR (Supplemental Table 1). Sequences were cloned into the BbsI site of pX330-U6 chimeric BB-CBh-hSpCas9 plasmid (42230; Addgene). HiScribe T7 mRNA synthesis kit (New England BioLabs) was used to in vitro transcribe the guide RNAs, and RNAs were subsequently purified using RNA Clean & Concentrator-5 (Zymo Research, Cat. R1016) before assessment on an Agilent Bioanalyzer as described (Han et al. 2015).

Super-ovulating 4–6-wk-old FVB/NJ female mice were mated with C57BL/6J males. After fertilization, the eggs were collected from the oviducts. Mouse zygotes were microinjected into the cytoplasm with Cas9 mRNA (100 ng/µL) and the gRNAs (100 ng/µL each) as described previously (Han et al. 2015; Oliver et al. 2015; Halim et al. 2017). After injection, zygotes were cultured in KSOM + AA medium (Millipore) for 1 h at 5% CO_2_ before transfer into the 7- to 10-wk-old female CD1 foster mothers.

Genomic DNA was isolated from tail-snips of founder mice by overnight proteinase K digestion. Two sets of primers flanking the deletion regions were used for PCR genotyping to detect the deletion using Taq Polymerase (NEB). Sibling founder mice were crossed together to generate the F1 generation. An F1 male harboring both the Deletion 2 and Deletion 3 alleles was crossed to C57BL/6 female mice to establish separate Deletion 2 and Deletion 3 lines. The F1 Deletion 1 animals were first crossed to heterozygous littermates (Del1/WT x Del1/WT). An F2 Del1 heterozygote was crossed to C57BL/6 female mice to establish the Deletion 1 line. All PCR products were resolved in 1% agarose gels. To confirm the deletions and check for any insertion events, Sanger sequencing of gel purified PCR products was performed (Nevada Genomics Center, University of Nevada). For the experiments in this paper, Deletion 3 line was backcrossed with C57BL/6 at least three times to stabilize the background, and heterozygote pairs were interbred to obtain *Calm1^+/+^* and *Calm1^ΔL/ΔL^* unless otherwise stated.

### Immunofluorescence

For whole-mount analysis, small tears were made in embryos in the forebrain and roof plate of the hindbrain to facilitate the penetration of PFA into the neural tube. Embryos were fixed by overnight incubation in 4% PFA at 4°C. Embryos were blocked and permeabilized for antibody labeling by performing 3 × 5 min, 3 × 30 min, and then overnight washes of PBST (10% FBS and 1% Triton X-100 in 1× PBS). Primary antibodies (Biolegend cat#801201 anti-β3Tubulin; 1:1000) were then added and incubated for two nights rocking at 4°C. Embryos were again washed for 3 × 5 min, 3 × 30 min, and then overnight in fresh PBST. Secondary antibodies were added at a 1:200 dilution for two nights at 4°C rocking in the dark. Secondary antibodies were washed off again 3 × 5 min, 3 × 30 min, and then overnight in fresh PBST. Embryos were then immersed in 80% glycerol/PBS for a minimum of 2 h, were mounted, and then imaged whole mount. A Leica SP8 TCS confocal microscope was used for imaging. For whole mount samples, images were taken at 1048 × 1048px, 200 Hz scanning speed, 3-line averaging, 8 bit, and with the use of linear-*z* compensation.

For free-floating brain section staining, PFA perfused brains were postfixed in 1% PFA for two nights and sectioned using vibratome. An amount of 80 µm thick sections were immediately used or stored at −20°C in antifreeze solution (30% w/v sucrose, 1% w/v PVP-40, 30% v/v ethylene glycol in PBS) until use. Sections were blocked in blocking solution for 2 h at RT, primary antibodies (cFos SYSY 226003; Npas4 Activity Signaling AS-AB18A) were incubated overnight at 4°C rocking in the dark. 10 min × 3 washes were performed in PBS followed by fluorophore labeled secondary antibody incubation at RT for 2 h. During 10 min × 3 washes, the nuclei were counterstained with DAPI. Sections were mounted in ProLong diamond antifade mountant (Invitrogen). Sections were cured at RT for at least 3 h and stored at 4°C until imaging. A Leica SP8 TCS confocal microscope was used for imaging at 1024 × 1024 px, 20× magnification, 3-line average and images were stitched automatically.

### DRG migration in vivo analysis

Whole mount E10.5 embryos were labeled with anti-β3Tubulin (Biolegend Cat# 801201 1:1000) and were subsequently imaged by confocal microscopy. Images were analyzed using FIJI/ImageJ (NIH). The cell bodies to be quantified were defined by drawing a line parallel to the last branch of the hypoglossal nerve extending across the length of the image. The hypoglossal nerve was chosen as an anatomical landmark because its morphology was unaffected in mutant embryos and served as an independent reference point so measurements were consistent across embryos. The area and distance migrated of cell bodies and axons rostral to the hypoglossal branch were measured using a freehand selection tool in FIJI/ImageJ. The multipoint cell counting tool was used on FIJI/ImageJ to count the number of axon bundles branching off the C1 DRG. A two-sided Student's *t*-test was performed to test for significance.

### Enriched environment-induced neural activation assay

Enriched environment (EE) was created in an 83.8 cm × 51.1 cm × H 34.3 cm box with sufficient bedding material at the bottom. Different types of toys were placed in the box with the sides of the box covered with colorful papers as illustrated in Figure 6A. Age matched *Calm1^+/+^* and *Calm1^ΔL/ΔL^* naïve animals (not used for other tests) were kept in their home cage undisturbed for at least 3 d prior to the experiment and transferred to the EE box on the test day. Mice were exposed to the EE for 1.5 h before euthanasia and tissue fixation. Brain sections were analyzed for IEG expression induction using free-floating immunofluorescence staining as described above. IEG positive cells were counted in the entire CA1 regions using FIJI/ImageJ. Images were adjusted for levels and color balance automatically and pixels were smoothened. Then, image thresholding was performed using Huang's method. After applying watershed function to separate closely located cells, particle analysis was performed in CA1 area to count cells with pixel units above 40. The parameters were set through repetitive rounds of visual inspection that allowed the most accurate detection of IEG positive cells and applied equally across all the images. The number of IEG positive cells were then compared to the total DAPI counts in the same CA1 region to estimate the percentage of activated neurons.

### Open-field assay

To assess locomotor function and exploratory behavior of *Calm1^+/+^* and *Calm1^ΔL/ΔL^* mice, open field assay was performed using ANY-maze video tracking system. The day before the assay, age-matched naïve animals were placed in the behavior room in their home cage for at least 30 min and gently handled for a couple of minutes to minimize distress of animals during the test. The animals were returned to the housing room. On the test day, the animals were brought in their home cages into the behavior room 30 min prior to starting the test. Overhead lighting setup was used to prevent shadows. A single animal was placed in the periphery of the arena facing the wall, and its behavior was recorded for 10 min. The apparatus was cleaned with 70% ethanol and dried for 5 min between each testing session. Total distance traveled by each mouse, mean speed, time spent in periphery, and exploration area were obtained to compare the behavior between *Calm1^+/+^* and *Calm1^ΔL/ΔL^* mice using ANY-maze software.

## SUPPLEMENTAL MATERIAL

Supplemental material is available for this article.
